# Facilitation in the soil microbiome does not necessarily lead to niche expansion

**DOI:** 10.1186/s40793-021-00373-2

**Published:** 2021-02-15

**Authors:** Xue Zhou, Márcio F. A. Leite, Zhenqing Zhang, Lei Tian, Jingjing Chang, Lina Ma, Xiujun Li, Johannes A. van Veen, Chunjie Tian, Eiko E. Kuramae

**Affiliations:** 1 College of Resources and Environment, Jilin Agricultural University, Changchun, China; 2 Key Laboratory of Mollisols Agroecology, Northeast Institute of Geography and Agroecology, Chinese Academy of Sciences, Changchun, China; 3 Department of Microbial Ecology, Netherlands Institute of Ecology NIOO-KNAW, Wageningen, the Netherlands; 4 Key Laboratory of Wetland Ecology and Environment, Northeast Institute of Geography and Agroecology, Chinese Academy of Sciences, Changchun, China; 5 University of the Chinese Academy of Sciences, Beijing, China; 6 Ecology and biodiversity, Institute of Environmental Biology, Utrecht University, Utrecht, The Netherlands

**Keywords:** Microbial co-occurrence, Facilitation, Stress gradient hypothesis, Latent variable modelling, C/N ratio, Elevation

## Abstract

**Background:**

The soil microbiome drives soil ecosystem function, and soil microbial functionality is directly linked to interactions between microbes and the soil environment. However, the context-dependent interactions in the soil microbiome remain largely unknown.

**Results:**

Using latent variable models (LVMs), we disentangle the biotic and abiotic interactions of soil bacteria, fungi and environmental factors using the Qinghai-Tibetan Plateau soil ecosystem as a model. Our results show that soil bacteria and fungi not only interact with each other but also shift from competition to facilitation or vice versa depending on environmental variation; that is, the nature of their interactions is context-dependent.

**Conclusions:**

Overall, elevation is the environmental gradient that most promotes facilitative interactions among microbes but is not a major driver of soil microbial community composition, as evidenced by variance partitioning. The larger the tolerance of a microbe to a specific environmental gradient, the lesser likely it is to interact with other soil microbes, which suggests that facilitation does not necessarily lead to niche expansion.

**Supplementary Information:**

The online version contains supplementary material available at 10.1186/s40793-021-00373-2.

## Background

Soil microbial communities are of vital importance to plant productivity, climate change and overall ecosystem functioning. Soil ecosystem functioning is the result of myriad interactions between microbes and soil environmental factors [1]. Many studies have sought to identify co-occurring microbes to explain their mutual interactions and correlations with different ecosystem services [2–4], while others have focused on understanding the role of soil factors in determining microbial community structure [5]. However, these studies have failed to discriminate direct microbial-microbial interactions from those induced by a third factor, such as soil factors, because the statistical methods adopted did not account for the influence of environmental factors in biasing the co-occurrences patterns. Moreover, environmental factors may also determine the interactions or not between organisms, the so-called context-dependent interactions [6]. These context-dependent interactions might determine the capability of the soil microbial community to deliver ecosystem services and thus influence overall ecosystem function [7], especially since soil factors are more important than land use in determining bacterial community structure [8], driving microbial community assembly [9] and influencing the plant response to inoculation, e.g., with mycorrhizal fungi [10]. The development of statistical methods that can detect biotic interactions of microorganisms and assess their influence in structuring microbial communities represents a challenge in microbial ecology.

Latent variable models (LVMs) offer the possibility of disentangling co-occurrence patterns into components describing shared environmental responses and residuals patterns of co-occurrence [11]. Thus, the microbial co-occurrence resulting from abiotic responses can be separated from the co-occurrence induced by biotic interactions [12]. LVMs can provide a better understanding of the ecological interactions of organisms [13, 14] and are increasingly being used in microbiome studies [15]. Moreover, LVMs can be used to explain changes in community abundance as a result of shifts in environmental factors. LVMs provide the highest posterior density (HPD) interval, a range of values in which an environmental variable determines the abundance of an organism, analogous to the confidence interval of a regression coefficient. Therefore, the HPD indicates the significance of an environmental variable (if the HPD interval contains zero, the influence of the variable is considered non-significant). In addition, the HPD interval indicates the tolerance or sensitivity of a specific organism to an environmental factor. The wider the HPD interval, the more tolerant an organism is to a specific environmental condition; if the interval includes zero, the organism does not depend on that environmental factor to exist. In summary, the HPD interval indicates the range of environmental conditions at which an organism might exist, thereby providing information on the organism’s multidimensional niche.

The aims of this study are to (1) disentangle the role of environmental factors in shifting interactions in the soil microbial community and (2) investigate the contribution of potential biotic interactions in determining the contraction or expansion of microbial niches by evaluating the relationship between the HPD interval and the residual correlations. We hypothesize that environmental factors shift not only the microbial community but also their interactions, which implies that the number and intensity of microbial interactions are related to the range of each covariate response. We test this hypothesis by investigating a gradient of soil environmental factors and the microbial community in the Qinghai-Tibetan Plateau wetland using LVMs. The Qinghai-Tibetan Plateau wetland, the largest and highest plateau on Earth, has suffered an unprecedented warming trend [16] that has reduced the area of Tibetan alpine tundra [17] and led to substantial changes in soil factors and soil microbial community, diversity, activity [16, 18] and functions [19]. Consequently, the Qinghai-Tibetan Plateau wetland is an interesting ecosystem to test our hypothesis.

## Results

### Soil physicochemical properties

The geographical and physicochemical characteristics of the 43 sites are summarized in Table S1. Soil TC varied greatly among the samples (from 3.25 to 345.69 g kg^− 1^). Soil pH varied from 6.42 to 9.25. Soil TN ranged from 0.58 to 17.35 g kg^− 1^. Soil TP varied from 0.32 to 2.13 g kg^− 1^. DOC ranged between 23.76 and 239.82 mg L^− 1^. The C/N ratio varied from 2.87 to 28.64. The E2/E3 ratio and SUVA254 ranged from 3.99 to 9.70 and 0.24 to 5.21, respectively. pH (*r* = − 0.551, *p* < 0.001), TN (*r* = 0.847, *p* < 0.001), DOC (*r* = 0.663, *p* < 0.001), TP (*r* = 0.572, *p* < 0.001) and C/N (*r* = 0.599, *p* < 0.001) were all significantly correlated with TC. However, the E2/E3 ratio (*r* = 0.039, *p* = 0.663) and SUVA254 (*r* = 0.029, *p* = 0.742) were not correlated with TC.

### Microbial community responses to environmental variation

The members of the microbial community responded differently to changes in pH, elevation, P, C, N, C/N, and the variables related to the characteristics of soil organic matter (DOC, E2/E3 ratio, and SUVA254). Supplementary Table S2 summarizes the number of microbial orders within each phylum that exhibited significant positive or negative coefficients for each environmental variable. To obtain an overview of the regression coefficients, we summarized those with significant values according to their median and interquantile range (IQR: the range of values corresponding to 50% of the total observed coefficients). This allowed us to better understand the intensity (median of coefficients) and direction of the effects (positive or negative) and general variability (IQR) for specific groups of microbes.

More bacterial (11) than fungal (6) orders increased in abundance with increasing elevation (median = + 0.83; IQR = [0.52 to 1.01]), and more fungal (6) than bacterial (4) orders decreased in abundance with increasing elevation (median = − 0.85; IQR = [− 1.18 to − 0.59]) (Fig. S1).

The abundances of a total of 39 bacterial and 6 fungal orders increased with increasing pH. More bacterial orders responded to increasing pH positively (39) than negatively (4), whereas more fungal orders responded negatively (8) than positively (6). *Capnodiales* (*Ascomycota*) had the largest positive coefficient (+ 1.96), whereas *Entorrhizales* (*Basidiomycota*) exhibited the smallest coefficient (− 3.17) (Supplementary Fig. S1). Similarly, changes in TC had more positive than negative effects on the bacterial community (Supplementary Fig. S2); 14 bacterial orders increased and five decreased in abundance with increasing TC (Supplementary Table S2). The response of the fungal community to TC was nearly opposite that of the bacterial community, as three and nine fungal orders responded positively and negatively, respectively. Although the number of microbes affected by TC was relatively small compared to the response to pH, the coefficient values were larger, ranging from − 3.60 to 4.17, suggesting that TC imposed stronger changes in relative abundance in the microbial community than pH.

The relative abundances of 34 microbial orders shifted in response to increasing TN: one bacterial and eight fungal orders responded positively (IQR = 0.60 to 1.81), whereas four bacterial and three fungal orders responded negatively (IQR = − 1.47 to − 0.82). The microbes belonged to seven different phyla (*Acidobacteria*, *Actinobacteria*, *Bacteroidetes*, *Chloroflexi*, *Firmicutes*, *Proteobacteria*, *Ascomycota*, *Basidiomycota*, and *Chytridiomycota*) (Supplementary Table S2).

Interestingly, the response to the C:N ratio (Supplementary Fig. S3) differed from the isolated effects of C and N. Thirteen bacterial and fungal orders from different phyla exhibited positive responses to increasing C:N: one *Bacteroidetes* (*Bacteroidales* order), one *Cyanobacteria*, five *Proteobacteria*, and five *Ascomycota*. Twenty-three bacterial orders from 8 different phyla (*Acidobacteria*, *Actinobacteria*, *Bacteroidetes*, *Chlorobi*, *Chloroflexi*, *Gemmatimonadetes*, *Nitrospirae*, and *Proteobacteria*) and four fungal orders responded negatively to an increasing C:N ratio. *Hysteriales* (*Ascomycota*) was most favored by increasing the C:N ratio (Supplementary Fig. S3). Similar to the C:N ratio, any increase in TP above its average value (1.15 g·kg^− 1^) reduced the abundances of bacteria (12) and fungi (5) (Supplementary Fig. S4). Their coefficients had IQRs of − 0.74 to − 0.44. Only one bacterial order, *Ktedonobacteria* C0119 (*Chloroflexi*), and three fungal orders, *Pleosporales*, *Pezizales* and *Dothideomycetes* (*Ascomycota*), showed a preference for higher values of TP (Supplementary Fig. S4).

High values of DOC increased the abundances of 8 bacterial and 8 fungal orders (Supplementary Fig. S3) distributed in six phyla (*Acidobacteria*, *Actinobacteria*, *Deinococcus*-*Thermus*, *Proteobacteria*, *Ascomycota*, and *Basidiomycota*). Their IQRs varied between 0.43 and 1.17. Only fungi belonging to *Pleosporales* seemed to prefer lower values of DOC, and seven orders belonging to the phyla *Actinobacteria*, *Aminicenantes*, *Bacteroidetes*, *Chloroflexi* and *Proteobacteria* preferred high DOC values. DOC molecular size also influenced the soil microbiome. Increases in the E2/E3 ratio negatively impacted 13 microbial orders from five different phyla (*Actinobacteria*, *Bacteroidetes*, *Chloroflexi*, *Proteobacteria*, and *Ascomycota*) (Supplementary Fig. S3). The coefficients ranged from − 2.41 to − 0.30. Only five fungal orders from three different phyla (*Ascomycota*, *Basidiomycota* and *Glomeromycota*) responded positively to changes in the E2/E3 ratio, with coefficients ranging from 0.59 to 1.18. By contrast, more orders were affected positively (33) than negatively (12) by SUVA254 (Supplementary Fig. S3). Most of the positive responders belonged to the phyla *Ascomycota* and *Proteobacteria* (7 orders), while the others belonged to six bacterial phyla (*Acidobacteria*, *Actinobacteria*, *Chloroflexi*, *Firmicutes*, *Gemmatimonadetes*, and *Nitrospirae*) and two fungal phyla (*Basidiomycota* and *Chytridiomycota*). The positive coefficients ranged from 3.12 to 0.30. The orders that decreased in abundance with increasing SUVA254 included one fungal order (*Pleosporales*) and 11 bacterial orders belonging to seven different phyla (*Acidobacteria*, *Actinobacteria*, *Aminicenantes*, *Bacteroidetes*, *Fibrobacteres*, *Firmicutes*, and *Proteobacteria*). The negative coefficients ranged from − 1.75 to − 0.44.

**Fig. 1:**
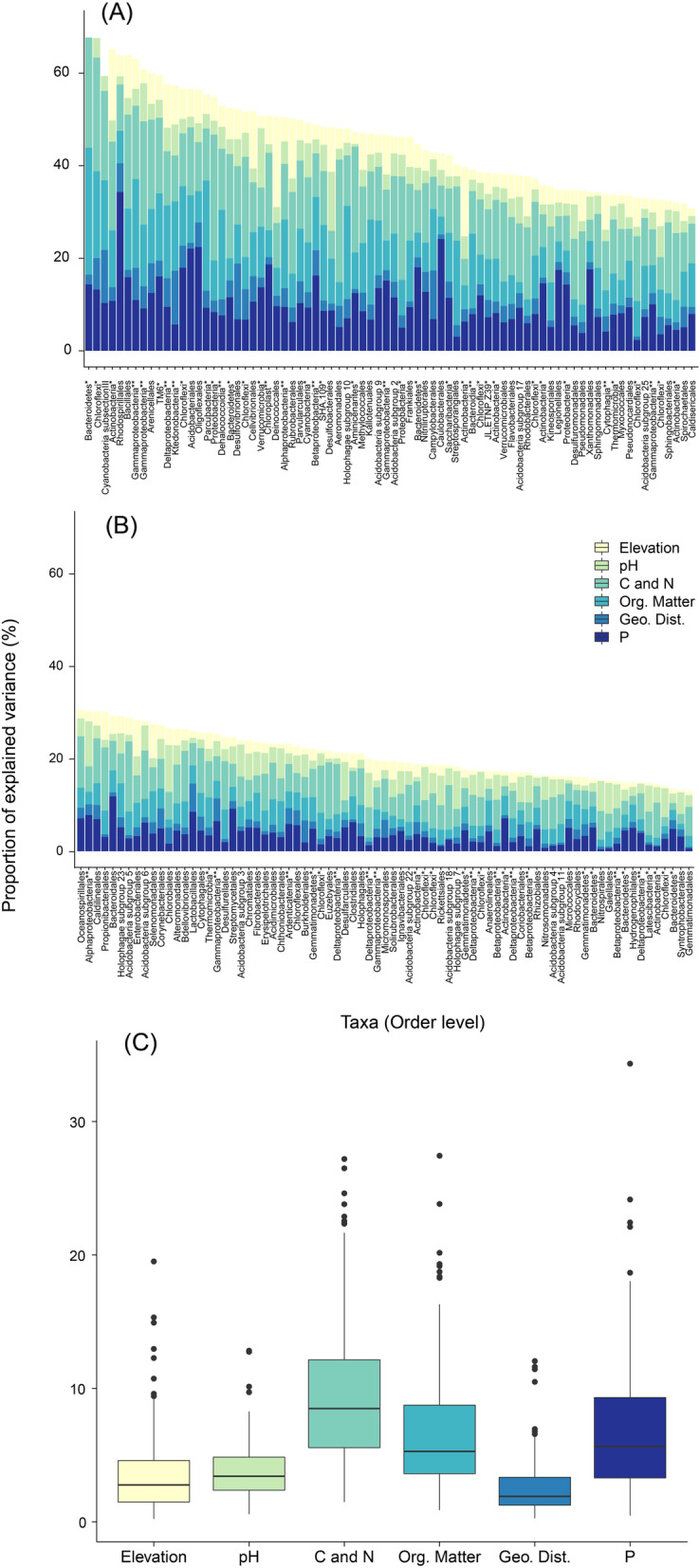
Analysis of variation partitioning showing the percentage of the variance of the bacterial community explained by elevation, pH, C and N (total C, total N, and C:N ratio), organic matter (DOC, E2/E3 ratio, and SUVA254), geographical distance (latitude and longitude) and P. (**A**) microbes with more than 30% of variance explained by the environmental factors; (**B**) microbes with less than 30% of variance explained by the environmental factors; (**C**) boxplot of all bacteria for each group of environmental variable. Taxa not classified at the order level are represented by * and ** for the phylum and class levels, respectively

We performed variance partitioning to determine how much of the variability of each order could be explained by a single or group of environmental factors using Boral analysis. The influence of environmental factors differed according to order for both the fungal and bacterial communities. Overall, the set of covariates (pH, elevation, P, C, N, C/N, DOC, E2/E3 ratio, SUVA254, and geographic distance) explained an average of 39.74% of soil microbial abundance, with a range of 80.13% (*Entorrhizales*) to 13.0% (*Gemmatimonadales*). The environmental variables explained more than 50% of the variability for only 27 bacterial orders (17.4%), as shown in Fig. 1, and on average the soil factors contributed to explaining 22.8 ± 10.22% of the bacterial abundance. The average influence of geographical distance on microbial variability was 7.06 ± 5.3%. C, N (TC, TN, and C:N ratio), P, and the organic matter characteristics (DOC, E2/E3, and SUVA254) contributed equally in determining the bacterial community. However, their influences were all significantly higher than the influence of elevation (median 3.58%; IQR 1.81–5.96%) and pH (median 3.79; IQR 2.57–5.38%) (Fig. 1). We included a measurement of skewness to illustrate the uneven distribution of the bacterial data compared with the fungal data. We also observed a highly positively skewed distribution of the proportion of variance (0.61), indicating that only a small part of the variation of the bacterial orders can be explained by considering only the measured covariates (Fig. 1).

**Fig. 2:**
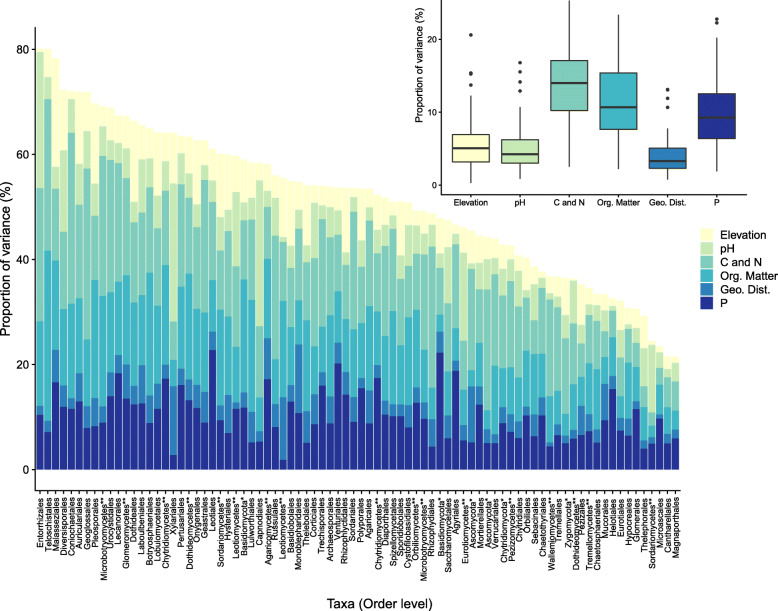
Analysis of variation partitioning showing the percentage of variance of the fungal community explained by elevation, pH, C and N (total C, total N, and C:N ratio), organic matter (DOC, E2/E3 ratio, and SUVA254), geographical distance (latitude and longitude) and P. Taxa not classified at the order level are represented by * and ** for the phylum and class levels, respectively

The response of the fungal community differed from that of the bacterial community, with a greater proportion of variability explained by the selected environmental variables (Fig. 2). Overall, the environmental factors explained more than 50% of the variability of 48 fungal orders, which represented 58.5% of the total evaluated fungi. The fungal community also exhibited greater sensitivity to changes in soil factors, which explained an average of 35.38 ± 11.8% of the variability. Consistent with the results for bacteria, the influence of geographic distance on the fungal community was minimal (10.0 ± 4.55%). We also observed a secondary role of changes in elevation in determining the fungal community, with an average explained variance of 5.79 ± 3.93%, similar to the influence of pH (5.75 ± 5.13%). For the fungal community, changes in C and N (14.34 ± 6.51%), organic matter (11.76 ± 5.66%), and P (10.01 ± 4.55%) exhibited the highest explanatory power. The fungal community also presented a less skewed distribution (− 0.17) than the bacterial community, indicating that the set of covariates played a major role in determining the fungal community, in contrast to the bacterial community (Fig. 2).

### Context-dependent interactions of the microbial community

The environmental factors not only contributed to shaping the microbial community but also shifted the strength of microbial interactions. We observed significant shifts in interactions between different microbial phyla (Table 1). Interestingly, changes in elevation produced the highest number of positive shifts in bacterial-bacterial interactions (Supplementary Fig. S5-S6). Changes in the C:N ratio induced more negative than positive shifts in both bacterial-bacterial and bacterial-fungal interactions (Supplementary Figure S7-S8). The responses to changes in pH were more equally distributed, with relatively equal numbers of positive and negative shifts. By comparison, fewer shifts were observed in the interactions between different fungi.

**Table 1:** Number of shifts in microbial interactions (positive or negative) in response to soil environmental factors (C:N ratio, elevation, and pH)

	C:N		Elevation		pH	
	Positive shift	Negative shift	Positive shift	Negative Shift	Positive shift	Negative shift
Bact-Bact	6	14	27	17	10	9
Fung-Fung	1	1	1	1	–	–
Bact-Fung	6	5	4	6	2	1

**Fig. 3:**
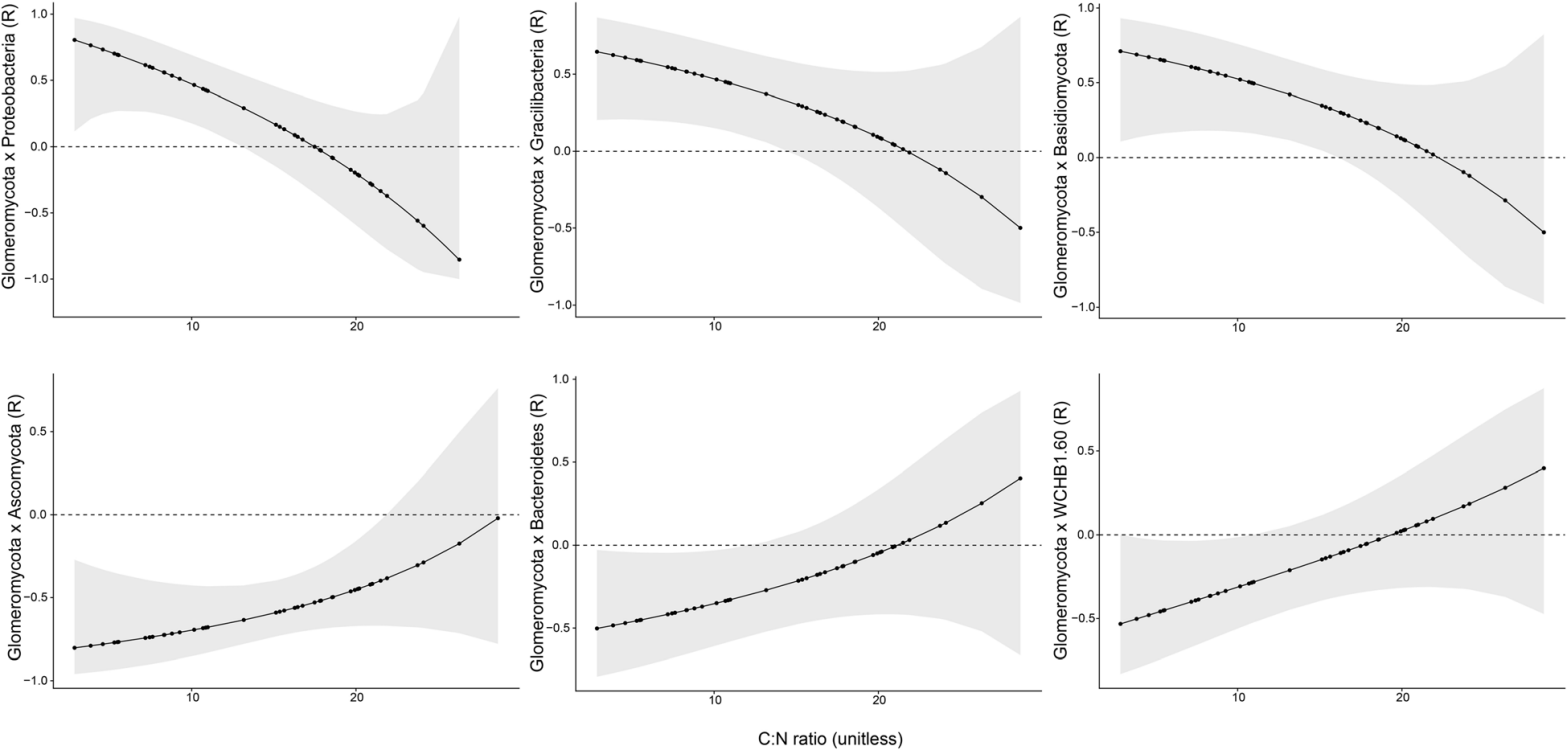
Significant shifts in correlation coefficients following changes in the C:N ratio between the fungal phylum *Glomeromycota* and the phyla *Proteobacteria*, *Gracilibacteria*, WCHB1–60, *Ascomycota*, *Bacteroidetes*, and *Basidiomycota*

The shifts in the interactions of the fungal phylum *Glomeromycota* (Fig. 3) in response to changes in the C:N ratio were particularly notable. This fungal phylum exhibited a significant positive shift when interacting with the bacterial phyla *Bacteroidetes* and WCHB1–60 and with the fungal phylum *Ascomycota*. By contrast, under an increasing C:N ratio, the phylum *Glomeromycota* appeared to shift from positive to negative interactions with the bacterial phyla *Proteobacteria* and *Gracilibacteria* and the fungal phylum *Basidiomycota*. The interactions with *Glomeromycota* were the only two significant shifts observed in response to changes in the C:N ratio (Table 1).

**Fig. 4:**
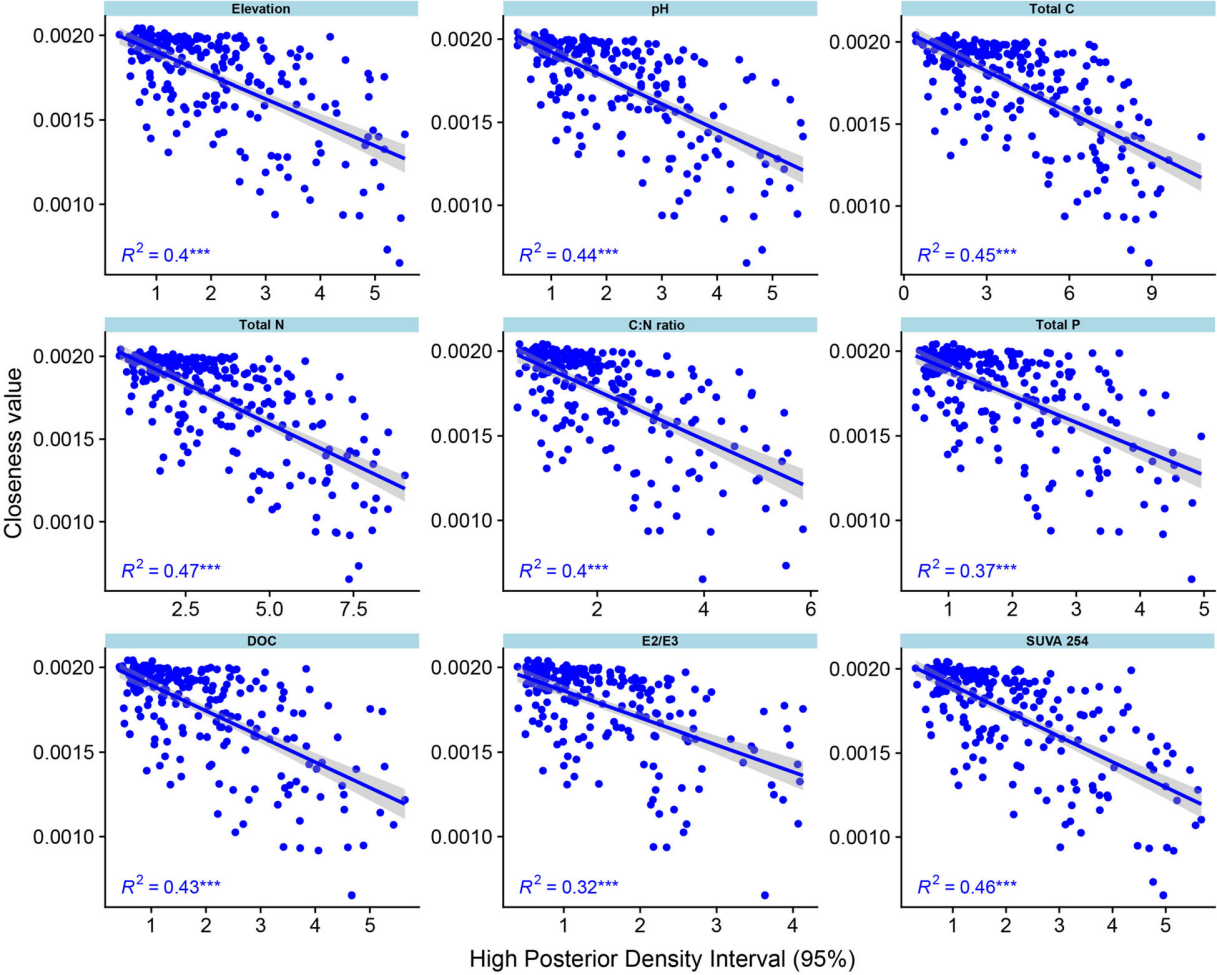
Relationship between the median of residual co-occurrence for each microbial taxon (order level) and the range of the high-density posterior interval (HPD interval) according to our standardized environmental factors (elevation and soil factors)

Remarkably, we also found a negative relationship between the closeness centrality measure and the range of environmental coefficients given by the HPD interval (Fig. 4) for all variables measured. In summary, the larger the HPD interval, the less likely a microbe is to co-occur with other microorganisms. Overall, the changes in the HPD interval explained 32% (E2/E3) to 47% (TN) of the variability in the closeness value.

## Discussion

### Microbial community shifts following changes in soil environmental factors

Our analysis provided many insights on the behavior of the soil microbial community under various environmental conditions. Overall, changes in pH influence more microbes than any other variable, but the proportion of variance explained by pH was smaller than that explained by the group of variables related to C and N (TC, TN, and C:N ratio), organic matter, and P. The majority of microbes preferred comparatively higher values of pH. The observed prevalence of positive coefficients for high pH suggests that the microbial community in the Qinghai-Tibetan plateau prefers more basic pH values.

Most of the soil microbes in this study preferred higher elevation, particularly bacteria, contrasting the findings in the Peruvian mountains [20]. We collected soil samples between 2717 and 4815 m a.s.l., with an average elevation of 3722 m a.s.l., whereas the samples collected in the Peruvian mountains ranged from 200 m to 3450 m a.s.l. from three different habitats (organic soil, mineral soil, and leaf surfaces). In our study, the populations of microbial taxa increased following the increase in elevation. This result suggests that we sampled an elevation gradient that significantly affected the microbial community, thereby providing a better understanding on the microbial community patterns at high elevation [21]. Yang et al. (2014) detected fewer genes at higher elevations than at 3400 m a.sl [22]., which they attributed to the presence of aboveground vegetation that could produce and supply organic C and other resources to the soil microbial community.

We found that the effects of the C:N ratio on the microbial community were more significant than the isolated effects of TC and TN. The C:N ratio of the studied sites ranged between 2.87 and 28.64. Increasing the C:N ratio reduced microbial populations due to the reduced availability of N, explaining the high proportion of negative coefficients. Our results indicate that the majority of these soil microbial communities preferred lower C:N ratios. Osler and Sommerkorn [23] introduced a framework suggesting that when the C:N ratios of microbial food resources are less than 25:1, there is an excess of N; however, at higher ratios, N becomes limiting, and microbes start to compete for N sources. Our results corroborate this framework by revealing that more bacteria were negatively than positively affected by high C:N ratio values. Interestingly, no significant reductions in the populations of fungal taxa (order level) were observed in our range of C:N ratios, likely due to the capacity of fungi to decompose more complex organic materials [24].

The changes in P content also explained a large proportion of the relative abundances of both bacteria and fungi. Overall, soil P content influenced more bacteria than fungi. Our results support the findings of Delgado-Baquerizo et al. [5], who demonstrated major roles of C, N, and P in shaping microbial community structure and extended their results by revealing a hierarchy of their influence on specific orders of both bacteria and fungi. Moreover, we also found a small role of pH in explaining microbial community variability. Since pH influences nutrient availability [25], the effect of pH likely represents an indirect measure of a nutrient not analyzed in this study. Such a possibility would explain the major role of pH in determining the microbial community in studies where pH is the only soil factor assessed [26]. Therefore, adding additional soil nutrient variables strongly impacts the explanatory power for microbial community relative abundance. Our study confirms the main role of soil nutrients in predicting microbial community structure and diversity [9, 27] and, by using a different approach, shows that bacterial abundance responds mainly to soil factors. Such a small and likely indirect role of pH is contrary to the current vision in soil microbiology.

The characteristics of soil organic matter also contributed to determining the microbial populations. The soil microbes preferred higher values of DOC and the low-molecular-weight fraction of organic matter, as reflected by a lower E2/E3 ratio. Some microbes also appeared to be favored by an increasing percentage of aromatic compounds in DOM, as reflected by the SUVA254 value.

### Context-dependent interactions of the soil microbiome

Although elevation did not have high explanatory power for microbial variation, it did result in more positive than negative shifts in microbial interactions. Our results extend to the microbial community the general notion that the importance of positive interactions increases under a stress gradient, as suggested by the stress gradient hypothesis (SGH) for plants [28]. According to He and Bertness [29], plant species interactions may shift from competition to facilitation with increasing stress. Based on our analysis, changes in elevation presented a gradient that promoted more positive microbial interactions. However, not every variable exhibited the same pattern. Increases in the C:N ratio resulted in more negative than positive shifts, suggesting increasing competition, whereas pH induced nearly equivalent numbers of positive and negative shifts. Therefore, our results indicate that while some environmental variables induce positive shifts in microbial interactions, others seem to promote increased competition. Microbial interactions respond to stress gradients, and elevation appears to be one type of stress. Nutrient limitation is a major factor controlling microbial activity. As the C:N ratio increases, microbes must compete for reduced N supply. Thus, the C:N ratio represents resource partitioning, in which microbes compete more strongly as nutrients become less available.

Interestingly, the interactions between different fungal phyla appeared more stable than the bacterial-bacterial and fungal-bacterial interactions (phylum level). However, the phylum *Glomeromycota* presented both positive and negative shifts. *Glomeromycota* is a new monophyletic phylum to which the mycorrhizal fungi were assigned [30]. These fungi possess an extensive external mycelium with phenotypic variation and may also interact with other soil microbes via the so-called mycorrhizosphere [31]. Previous research has shown that interactions with arbuscular mycorrhiza are context-dependent and likely determined by soil conditions [32]. Our results contribute to the understanding of soil mycorrhizal interactions by showing that the C:N ratio influences the strength of soil mycorrhizal associations not only with plants but also with other soil fungi.

The current literature divides the SGH into two different phenomena: (i) a shift from negative to positive interactions under a stress condition and (ii) niche expansion due to increased facilitation (positive interactions). Although we found evidence of positive shifts with increasing elevation, we observed a negative association between the centrality measures of our network of microbial co-occurrences and the microbial population range (given by the HPD interval). Thus, the larger the tolerance of a microbe to a given environmental variable, the less likely it is to interact with other microbes, as indicated by loss of centrality within the network. This relationship is consistent with the ecological phenomenon that a microbe that is more tolerant to or capable of growth under different environmental conditions is less likely to depend on interactions with other organisms. Such organisms tend toward neutrality. By contrast, the more sensitive a microbial population is to a small range of an environmental gradient, the more likely it is to depend on other microbes or compete against them.

The soil microbial communities of the Qinghai-Tibet Plateau are strongly influenced by the changes in soil organic matter, a soil variable previously reported as sensitive to ongoing climate warming [33]. Wang et al. [34] reported that warming in the Qinghai-Tibet Plateau promotes plants with less branches and thin roots resulting in the reduction of the median of root lifespan, and Jia et al. [33] showed that changes in temperature increased the soil organic matter turnover. In the current study, the microbial community presented great dependence on the organic matter that is mainly provided by the plants. Therefore, the ongoing climatic changes might affect the plant physiology and consequently the soil organic matter. Altogether, the climatic changes can increase the selection pressure on microbes through changes in the soil organic matter. Previous studies have shown that warming impacts the soil microbial community structure by favoring more fungal than bacterial groups [16]. Since no evidence of niche expansion was found in the current study, some soil microbes might not be able to adapt to the warming trend in Qinghai-Tibet Plateau. A deeper understanding of the effects of global warming is left as an avenue for future research.

## Conclusions

In summary, our results show that the members of the soil microbiome not only interact with each other but also shift from competition to facilitation or from facilitation to competition depending on the environmental variable, giving rise to context-dependent interactions. Our findings indicate that the larger the tolerance of a microbe to some environmental gradient, the less likely it is to interact with other soil microbes. Overall, environmental gradients of elevation appear to promote more facilitative interactions among microbes, consistent with results for plants [35]. Furthermore, the shift toward more positive interactions does not necessarily lead to niche expansion.

## Methods

### Site description and soil sampling

**Fig. 5:**
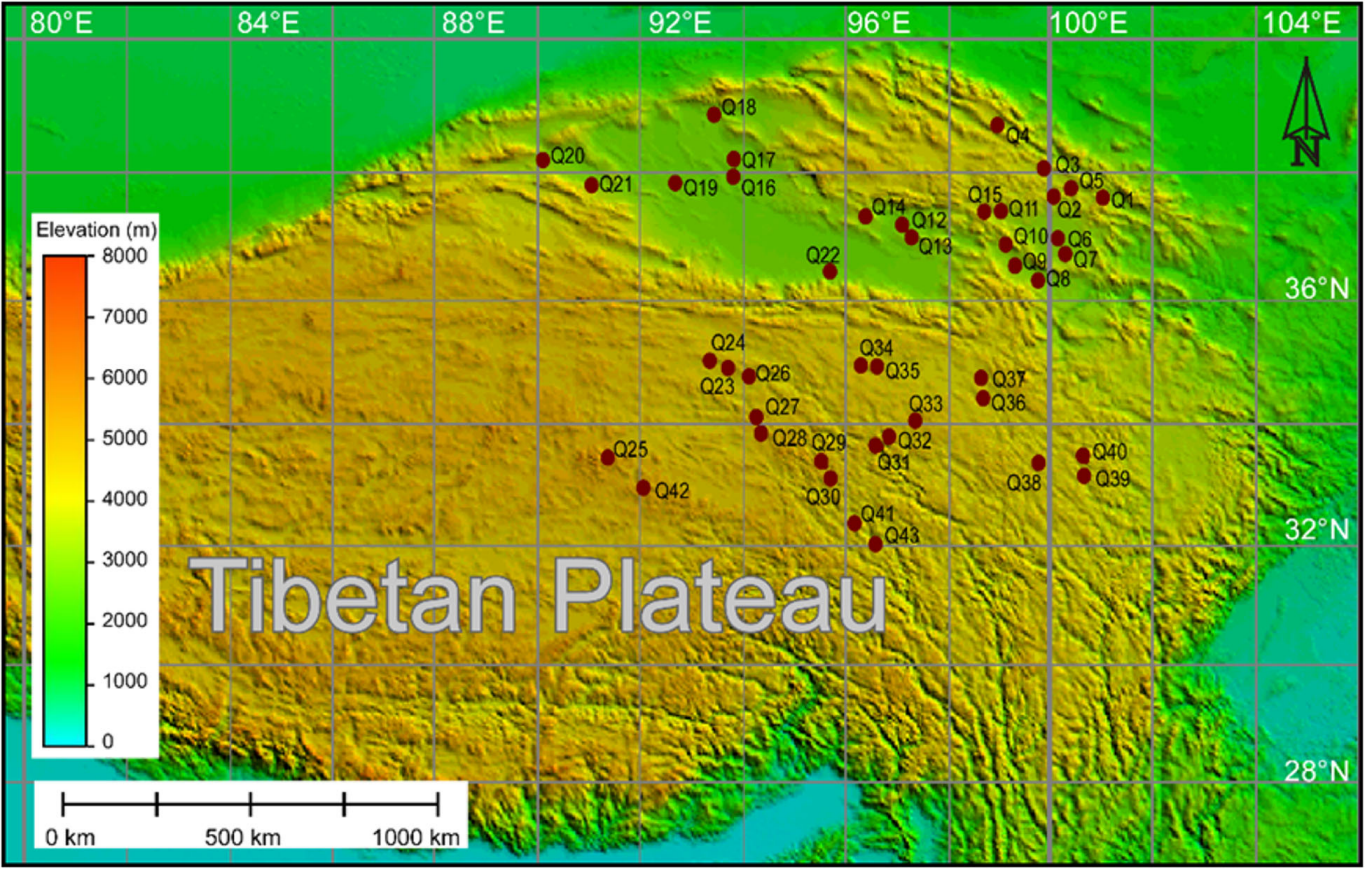
The sampling site in the northwestern part of the Qinghai-Tibetan Plateau at elevations ranging from 2717 to 4815 m above sea level and a longitude (90° to 102°) and latitude (32° to 39°) gradient covering the entire natural wetland area of Qinghai Province

The sampling area is in the northwestern part of the Qinghai-Tibetan Plateau at elevations ranging from 2717 to 4815 m above sea level and has a longitudinal (90° to 102°) and latitudinal (32° to 39°) gradient covering the entire natural wetland area of Qinghai Province. The Qinghai-Tibetan Plateau has a continental high-plateau monsoon climate characterized by long, cold winters and short, warm summers. The mean annual air temperature ranges from − 4 °C to 8 °C, and the average annual precipitation is 650 mm [36]. The vegetation cover is primarily *Carex meyeriana* and *Carex muliensis*. Due to the alpine environment, the plant communities have a short growth period and low primary production and diversity. In July 2015, soil samples were collected from 43 sites (Fig. 5). Five soil cores with a diameter of 1.5 cm were randomly taken at a depth of 0–30 cm from each site. The samples were pooled to one sample per site following the same procedures as in Kuramae et al. [27] in order to avoid the bias from small scale soil variability and to obtain a more representative sample of the site. The soil samples were transported to the laboratory on ice and sieved with a 2-mm mesh to remove roots and stones. Soil samples for soil physicochemical characterization and DNA extraction were preserved at − 4 °C and − 80 °C, respectively.

### Soil physicochemical characterization

Soil pH was measured in soil-water suspensions (1:5, w/w) [37]. The total nitrogen (TN) in the soil was determined by dichromate oxidation using a continuous flow analytical system (SAN++, SKALAR, Netherlands). The total phosphorus (TP) in the soil was determined by the HClO_4_-H_2_SO_4_ digestion method according to the soil analysis manual [38, 39]. The potassium dichromate oxidation method was used to analyze SOC [40]. The dissolved organic matter (DOM) fraction of the soil samples was extracted with deionized water (solid-to-water ratio of 1:2.5 w/v). Fresh soil samples were added to deionized water and shaken for 24 h in a horizontal shaker at room temperature. The DOM extract was filtered using 0.45-μm membrane filters and further diluted before measurement in a multi-mode microplate reader (Synergy H1, BioTek). The spectra were blank-corrected with distilled water. Each sample was scanned eight times, and the average was reported. Two optical properties (the E2/E3 ratio and specific UV absorbance at 254 nm (SUVA254)) were measured to assess different DOM properties. The E2/E3 ratio of the absorbance at 250 to 365 nm was used to estimate DOM molecular size. As high-molecular-weight DOM absorbs at the longer wavelength, the E2/E3 ratio is lower when the high-molecular-weight DOM fraction is greater [41]. SUVA254, which correlates with the fraction of aromatic compounds in DOM [42], was calculated by dividing the UV absorbance at 254 nm by the concentration of DOC.

### DNA extraction and sequencing

Genomic DNA was extracted from 0.5 g of soil using a FASTDNA™ SPIN Kit for soil (MPBio, Santa Ana, CA, USA) according to the manufacturer’s instructions. The DNA concentration was measured using a NanoDrop 2000 spectrophotometer (NanoDrop Technologies, Inc., Wilmington, DE, USA). The V3-V4 regions of the bacterial 16S rRNA gene and the fungal ITS2 region were amplified using the primer pair 341F (CCTAYGGGRBGCASCAG) and 806R (GGACTACNNGGGTATCTAAT) and the primer pair ITS3F (GCATCGATGAAGAACGCAGC) and ITS4R (TCCTCCGCTTATTGATATGC) combined with Illumina adapter sequences, a pad and a linker of two bases and barcodes on the reverse primers [43]. The PCR reactions were performed in a 30-μL mixture containing 3 μL of each primer (2 μM), 10 μL of template DNA (1 ng/μL), 15 μL of Phusion® High-Fidelity PCR Master Mix (New England BioLabs, Inc., Ipswich, MA, USA) and 2 μL of water. The following thermal program was used for amplification: 95 °C for 1 min, followed by 30 cycles of 98 °C for 10 s, 50 °C for 30 s, and 72 °C for 30 s and a final extension step at 72 °C for 5 min. Each sample was amplified in triplicate, and the PCR products were pooled and purified using a Qiagen Gel Extraction Kit (Qiagen, Hilden, Germany). Sequencing libraries were generated using a TruSeq® DNA PCR Free Sample Preparation Kit (Illumina, San Diego, CA, USA) according to the manufacturer’s instructions and pooled at an equimolar ratio. An Illumina HiSeq2000 platform at Novogene Bioinformatics Technology Ltd., Beijing, China, was used to perform 250-bp paired-end sequencing. The raw sequence datasets were deposited in the NCBI SRA database under accession number SRP158093.

### Amplicon sequence analysis

Raw sequences were divided into sample libraries via sample-specific barcodes and truncated after cutting off the barcode and primer sequences. Forward and reverse reads with at least 10 bp of overlap and less than 5% mismatch were merged using FLASH [44]. Quality filtering on the raw tags was performed according to the QIIME (V1.7.0, http://qiime.org/index.html) quality control process [45], and all sequences shorter than 200 bp or with an average quality score of less than 25 were removed from the raw reads. The remaining sequences were subjected to chimera removal using the UCHIME Algorithm (http://www.drive5.com/usearch/manual/uchime_algo.html). Uparse (Version 7.0.1001, http://drive5.com/uparse/) was used to classify the operational taxonomic units (OTUs) at the 97% similarity level [46]. The longest sequence with the greatest number of hits to other sequences in each OTU was screened as a representative sequence. All OTUs with sequence numbers ≤2 were removed in subsequent analyses. For ITS sequences, the taxonomic identity was annotated by QIIME software using a Blast algorithm against sequences in the Unite Database (https://unite.ut.ee/); for 16S rRNA sequences, the taxonomic classification was based on the SILVA Database (http://www.arb-silva.de/) in Mothur [47].

### Statistical analysis

To assess the influence of environmental variables (elevation, pH, total carbon (TC), TN, C:N ratio, TP, DOC, E2/E3 ratio, SUVA254) and geographic distance (because the sampling points differ in distance, part of the microbial community variability is a result of geographic distance) on the microbial communities, we used Latent Variable Models (LVMs) [14] provided by the boral package of R [48]. Boral package provides model-based ordination using Bayesian statistics to capture the sources of variability in the abundance of different organisms while account for the effect of environmental variables and co-occurrences [12]. The advantage of using LVM is the capacity to quantify to what extent the variations of bacterial and fungal order taxonomic level were explained by environmental covariates (elevation and soil factors) and geographic distance; thus, disentangling the sources of variability in the microbial communities and allowing us to better identify the microbial responses to changes in elevation and soil factors. Studying the microbial community composition at high taxonomic resolution based solely on short reads is challenging, and the choice of taxonomic levels depends on the quality of the sequence and is limited by available information in reference databases. The accuracy of LVM estimates is reduced for low-occurring organisms, and analyses of rare microbes (i.e., microbes occurring in less than five samples) are unreliable. Because the proportion of low-occurring microbes increases greatly at higher taxonomic levels, LVM adoption would require the removal of those low-occurring microbes and limit the analysis of potential biotic interactions. Therefore, to provide a clear but detailed analysis and avoid overinterpretation at the OTU level, all analyses of bacterial and fungal communities were performed at the Order taxonomic level and all the taxa that occurred in less than five samples were removed. The models were fit assuming weakly informative priors [49]. Priors are probability values used as the starting point of Bayesian analysis; the choice of weakly informative priors reduces bias in the analysis. According to [50], microbiome sequencing data are compositional. To account for that compositional nature, we included a sample effect in the LVM. In addition, differences in the scales of soil factor variables (g/cm^3^, mg/dm^3^, etc.) could bias the analysis of variance partitioning. Consequently, to guarantee an even comparison in the analysis of variance partitioning, we standardized all environmental factors to units of standard deviation from the mean of each environmental variable.

As stated earlier, LVMs are joint models [14], thus, we were able to evaluate not only the changes in abundance induced by environmental factors but also the co-occurrence between organisms. Then, for the microbial community, LVM provides regression coefficients that described the influence of each covariate (soil factors, elevation, and geographical distance) on the microbial relative abundance and co-occurrences between the different microbial populations. To determine the significance of each regression coefficient, we checked whether or not the HPD interval included zero. To summarize all the significant effects, we evaluated the median and interquartile range (IQR). This analysis allowed us to understand the strength of the influence of each environmental variable while grouping positive and negative effects at the phylum level.

The HPD interval not only informs whether a regression coefficient is significant but also reflects the range of an environmental factor in which a taxon occurs. Given our sampling size (45 sites), we also evaluated the HPD value as an estimate of the range of an environmental variable at which an organism might exist, thereby providing information on the organism’s multidimensional niche.

As a joint model, an LVM allows co-occurrences as a result of a shared environmental response to be disentangled from the residual correlation. The shared environmental response reflects any co-occurrence resulting from the influence of an environmental factor (e.g., soil nutrients, climate). Any other correlation that cannot be explained by any of our selected environmental factors is a residual correlation and can be considered a potential biotic interaction [14]. To investigate the contribution of biotic interactions in determining the contraction or expansion of microbial niches (order level), we combined the results obtained from the residual co-occurrence analysis with the values of the HPD interval. To do that, we evaluated the association between the HPD interval and the co-occurrence network from the residual correlation. We selected the closeness values as an indicator of the co-occurrence network. The closeness value is a measure of node centrality and thus indicates how dependent each microbe is on all others. We hypothesized that the dependence of a microbe (measured by its closeness) is related to its niche (measured by the HPD interval).

Since part of the microbial co-occurrence is a result of the shared environmental response, this co-occurrence can shift from positive to negative or vice versa as a result of changes in the environmental variables. To investigate the changes in microbial community interactions, we used a conditional estimator for Spearman’s correlation between two different microbial phyla (fungi and bacteria) adjusted for the environmental variables of elevation, C:N ratio and pH according to the method developed by Liu et al. [51]. This method uses a semiparametric cumulative probability model to preserve the rank-based nature of Spearman’s correlation while handling the overdispersed nature of the data. Due to the limited number of samples in the face of the high number of possible combinations of interactions, we performed this analysis at the phylum level.

## Supplementary Information

**Additional file 1.**

## Data Availability

The datasets (raw sequences) generated and analysed during the current study were deposited in the NCBI SRA database repository, under the accession number SRP158093 and will be available once the manuscript is accepted for publication.

## References

[1] Bardgett RD, Freeman C, Ostle NJ (2008). Microbial contributions to climate change through carbon cycle feedbacks. ISME J.

[2] de Boer W (2017). Upscaling of fungal–bacterial interactions: from the lab to the field. Curr Opin Microbiol.

[3] Ho A, Angel R, Veraart AJ, Daebeler A, Jia Z, Kim S, Kerckhof F-M, Boon N, Bodelier PLE (2016). Biotic interactions in microbial communities as modulators of biogeochemical processes: Methanotrophy as a model system. Front Microbiol.

[4] Hoppe B, Kahl T, Karasch P, Wubet T, Bauhus J, Buscot F, Krüger D (2014). Network analysis reveals ecological links between N-fixing bacteria and wood-decaying fungi. PLoS One.

[5] Delgado-Baquerizo M, Reich PB, Khachane AN, Campbell CD, Thomas N, Freitag TE, Abu Al-Soud W, Sørensen S, Bardgett RD, Singh BK (2017). It is elemental: soil nutrient stoichiometry drives bacterial diversity. Environ Microbiol.

[6] Chamberlain SA, Bronstein JL, Rudgers JA (2014). How context dependent are species interactions?. Ecol Lett.

[7] Alzarhani KA, Clark DR, Underwood GJC, Ford H, Cotton ATE, Dumbrell AJ (2019). Are drivers of root-associated fungal community structure context specific?. ISME J.

[8] Kuramae EE, Yergeau E, Wong LC, Pijl AS, van Veen JA, Kowalchuk GA (2012). Soil characteristics more strongly influence soil bacterial communities than land-use type.

[9] Yan Y, Klinkhamer PGL, van Veen JA, Kuramaea EE (2019). Environmental filtering: A case of bacterial community assembly in soil. Soil Biol Biochem.

[10] Hoeksema JD, Chaudhary VB, Gehring CA, Johnson NC, Karst J, Koide RT, Pringle A, Zabinski C, Bever JD, Moore JC (2010). A meta-analysis of context-dependency in plant response to inoculation with mycorrhizal fungi. Ecol Lett.

[11] Pollock LJ, Tingley R, Morris WK, Golding N, O'Hara RB, Parris KM, Vesk PA, McCarthy MA (2014). Understanding co-occurrence by modelling species simultaneously with a joint species distribution model (JSDM). Methods Ecol Evol.

[12] Leite MF, Kuramae EE (2020). You must choose, but choose wisely: model-based approaches for microbial community analysis. Soil Biol Biochem.

[13] Wang X, Hua F, Wang L, Wilcove DS, Yu DW (2019). The biodiversity benefit of native forests and mixed-species plantations over monoculture plantations. Divers Distrib.

[14] Warton DI, Blanchet GF, O’Hara RB, Ovaskainen O, Taskinen S, Walker SC, Hui F (2015). So many variables: joint modeling in community ecology. Trends Ecol Evol.

[15] Sankaran K, Holmes SP (2018). Latent variable modeling for the microbiome. Biostatistics.

[16] Wang C, Zhao X, Zi H, Hu L, Ade L, Wang G, Lerdau M (2017). The effect of simulated warming on root dynamics and soil microbial community in an alpine meadow of the Qinghai-Tibet plateau. Appl Soil Ecol.

[17] Walker MD, Gould WA, Iii FSC (2001). Scenarios of biodiversity changes in arctic and alpine tundra.

[18] Zi HB, Hu L, Wang CT, Wang GX, Wu PF, Lerdau M, Ade LJ (2018). Responses of soil bacterial community and enzyme activity to experimental warming of an alpine meadow. Eur J Soil Sci.

[19] Zhao N, Zhang HX, Wang RM, Yang MY, Zhang Y, Zhao XN, Yu GR, He NP (2014). Effect of grazing intensity on temperature sensitivity of soil nitrogen mineralization in Zoige alpine meadow. Acta Ecol Sin.

[20] Fierer N, McCain CM, Meir P, Zimmermann M, Rapp JM, Silman MR, Knight R (2011). Microbes do not follow the elevational diversity patterns of plants and animals. Ecology.

[21] Noah F, Mccain CM, Patrick M, Michael Z, Rapp JM, Silman MR, Rob K (2011). Microbes do not follow the elevational diversity patterns of plants and animals. Ecology.

[22] Yang Y, Gao Y, Wang S, Xu D, Yu H, Wu L, Lin Q, Hu Y, Li X, He Z (2014). The microbial gene diversity along an elevation gradient of the Tibetan grassland. ISME J.

[23] Osler GHR, Sommerkorn M (2007). Toward a complete soil C and N cycle: incorporating the soil fauna. Ecology.

[24] Leite MFA, Pan Y, Bloem J, Berge H, Kuramae EE (2017). Organic nitrogen rearranges both structure and activity of the soil-borne microbial seedbank. Sci Rep.

[25] Navarrete AA, Tsai SM, Mendes LW, Karoline F, Mattias DH, Cassman NA, Jeroen R, van Veen JA, Kuramae EE (2015). Soil microbiome responses to the short-term effects of Amazonian deforestation. Mol Ecol.

[26] Fierer N, Jackson RB (2006). The diversity and biogeography of soil bacterial communities. Proc Natl Acad Sci U S A.

[27] Kuramae EE, Yergeau E, Wong LC, Pijl AS, Veen JA, Kowalchuk GA (2012). Soil characteristics more strongly influence soil bacterial communities than land-use type. FEMS Microbiol Ecol.

[28] He Q, Bertness MD, Altieri AH (2013). Global shifts towards positive species interactions with increasing environmental stress. Ecol Lett.

[29] He Q, Bertness MD (2014). Extreme stresses, niches, and positive species interactions along stress gradients. Ecology.

[30] Schüβler A, Schwarzott D, Walker C (2001). A new fungal phylum, the Glomeromycota: phylogeny and evolution* *dedicated to Manfred Kluge (Technische Universität Darmstadt) on the occasion of his retirement. Mycol Res.

[31] Johansson JF, Paul LR, Finlay RD (2004). Microbial interactions in the mycorrhizosphere and their significance for sustainable agriculture. FEMS Microbiol Ecol.

[32] Santos-González JC, Nallanchakravarthula S, Alström S, Finlay RD (2011). Soil, but not cultivar, shapes the structure of arbuscular mycorrhizal fungal assemblages associated with strawberry. Microb Ecol.

[33] Jia Y, Kuzyakov Y, Wang G, Tan W, Zhu B, Feng X (2020). Temperature sensitivity of decomposition of soil organic matter fractions increases with their turnover time. Land Degrad Dev.

[34] Wang Z, Ding L, Wang J, Zuo X, Yao S, Feng J (2016). Effects of root diameter, branch order, root depth, season and warming on root longevity in an alpine meadow. Ecol Res.

[35] Callaway RM, Brooker RW, Choler P, Kikvidze Z, Lortie CJ, Michalet R, Paolini L, Pugnaire FI, Newingham B, Aschehoug ET (2002). Positive interactions among alpine plants increase with stress. Nature.

[36] Chen GC, Huang ZW, Xue Feng LU, Peng M (2002). Characteristics of wetland and its conservation in the Qinghai plateau. J Glaciol Geocryol.

[37] Zhang B, Chen S, He X, Liu W, Zhao Q, Zhao L, Tian C (2014). Responses of soil microbial communities to experimental warming in Alpine grasslands on the Qinghai-Tibet plateau. PLoS One.

[38] Lao JC (1988). Handbook of soil chemical analysis.

[39] Chang C, Chen W, Luo S, Ma L, Li X, Tian C (2018). Rhizosphere microbiota assemblage associated with wild and cultivated soybeans grown in three types of soil suspensions. Arch Agron Soil Sci.

[40] Li X, Xue Z, Gao J (2016). Environmental influence on vegetation properties of frigid wetlands on the Qinghai-Tibet plateau, Western China. Wetlands.

[41] Weishaar JL, Aiken GR, Bergamaschi BA, Fram MS, Fujii R, Mopper K (2017). Evaluation of specific ultraviolet absorbance as an indicator of the chemical composition and reactivity of dissolved organic carbon. Environ Sci Technol.

[42] Tfaily MM, Podgorski DC, Corbett JE, Chanton JP, Cooper WT (2011). Influence of acidification on the optical properties and molecular composition of dissolved organic matter. Anal Chim Acta.

[43] Caporaso JG, Lauber CL, Walters WA, Berg-Lyons D, Huntley J, Fierer N, Owens SM, Betley J, Fraser L, Bauer M (2012). Ultra-high-throughput microbial community analysis on the Illumina HiSeq and MiSeq platforms. ISME J.

[44] Deng J, Gu Y, Zhang J, Xue K, Qin Y, Yuan M, Yin H, He Z, Wu L, Schuur EA (2015). Shifts of tundra bacterial and archaeal communities along a permafrost thaw gradient in Alaska. Mol Ecol.

[45] Bokulich NA, Subramanian S, Faith JJ, Gevers D, Gordon JI, Knight R, Mills DA, Caporaso JG (2013). Quality-filtering vastly improves diversity estimates from Illumina amplicon sequencing. Nat Methods.

[46] Edgar RC (2004). MUSCLE: multiple sequence alignment with high accuracy and high throughput. Nucleic Acids Res.

[47] Quast C, Pruesse E, Yilmaz P, Gerken J, Schweer T, Yarza P, Peplies J, Glöckner FO (2013). The SILVA ribosomal RNA gene database project: improved data processing and web-based tools. Nucleic Acids Res.

[48] Hui FKC (2016). Boral – Bayesian ordination and regression analysis of multivariate abundance data in r. Methods Ecol Evol.

[49] Gelman A, Jakulin A, Pittau MG, Su Y-S (2008). A weakly informative default prior distribution for logistic and other regression models. Ann Appl Stat.

[50] Gloor GB, Macklaim JM, Pawlowsky-Glahn V, Egozcue JJ (2017). Microbiome datasets are compositional: and this is not optional. Front Microbiol.

[51] Liu Q, Li C, Wanga V, Shepherd BE. Covariate-adjusted Spearman’s rank correlation with probability-scale residuals. Biometrics. 2017. 10.1111/biom.12812 PMC5949238 29131931

