# Supplementary material for: Facilitation in the soil microbiome does not necessarily lead to niche expansion
Source: Environ Microbiome. 2021 Feb 15;16:4. doi: 10.1186/s40793-021-00373-2 (PMC8067652; doi:10.1186/s40793-021-00373-2)
Supplement: Supplementary file 1 — Additional file 1. [file 40793_2021_373_MOESM1_ESM.docx]

**Facilitation in the soil microbiome does not necessarily lead to niche expansion**

Xue Zhou^1,2#^, Márcio F. A. Leite^3#^, Zhenqing Zhang^4^, Lei Tian ^1,5^, Lina Ma^1,5^, Xiujun Li^1^, Johannes A. van Veen^1,3^, Chunjie Tian^1*,^ Eiko E. Kuramae^3,6*^

^1^ Key Laboratory of Mollisols Agroecology, Northeast Institute of Geography and Agroecology, Chinese Academy of Sciences, Changchun, China

^2^ College of Resources and Environment, Jilin Agricultural University, Changchun, China

^3^ Department of Microbial Ecology, Netherlands Institute of Ecology NIOO-KNAW, Wageningen, the Netherlands

^4^ Key Laboratory of Wetland Ecology and Environment, Northeast Institute of Geography and Agroecology, Chinese Academy of Sciences, Changchun, China

^5^ University of the Chinese Academy of Sciences, Beijing, China

^6^ Ecology and biodiversity, Institute of Environmental Biology, Utrecht University, Utrecht, The Netherlands

#equal contribution

*Corresponding authors:

Eiko E. Kuramae, Department of Microbial Ecology, Netherlands Institute of Ecology NIOO-KNAW, Wageningen, The Netherlands, E-mail: [e.kuramae@nioo.knaw.nl](mailto:e.kuramae@nioo.knaw.nl)

Chunjie Tian, Key Laboratory of Mollisols Agroecology, Northeast Institute of Geography and Agroecology, Chinese Academy of Sciences, Changchun, 130102, China, E-mail: [Tiancj@iga.ac.cn](mailto:Tiancj@iga.ac.cn)

Running title: Soil microbial community interactions are context dependent

Keywords: Competition, Facilitation, Stress gradient hypothesis, Microbial co-occurrence, Latent variable modelling, C/N ratio, elevation.

**Supplementary Material**

Table S1. Sampling sites and soil properties.

| Sites | elevation | longitude | latitude | pH | TN  (g·kg^-1^) | TC  (g·kg^-1^) | DOC | C/N | E2/E3 | SUVA254 |
| --- | --- | --- | --- | --- | --- | --- | --- | --- | --- | --- |
| Q1 | 3210.00 | 101.34772° | 37.60166° | 8.89±0.47 | 3.46±0.18 | 13.14±5.37 | 66.23±8.74 | 3.85±1.77 | 4.51±0.06 | 1.77±0.37 |
| Q2 | 3510.00 | 100.67284° | 37.71442° | 7.34±0.43 | 10.25±0.70 | 111.01±6.62 | 86.73±13.09 | 10.86±0.92 | 4.86±0.01 | 3.11±0.45 |
| Q3 | 3764.00 | 100.28139° | 37.97375° | 7.27±0.38 | 10.87±1.29 | 116.73±22.64 | 125.27±34.38 | 10.69±0.91 | 4.21±0.20 | 1.74±0.08 |
| Q4 | 3499.00 | 99.28105° | 38.67726° | 6.89±0.04 | 7.76±0.62 | 78.80±10.77 | 28.75±6.03 | 10.15±1.03 | 4.54±0.03 | 2.91±0.72 |
| Q5 | 3658.00 | 100.40408° | 37.65521° | 7.94±0.18 | 7.04±0.74 | 32.35±4.93 | 47.49±0.77 | 4.59±0.45 | 4.83±0.23 | 4.17±0.15 |
| Q6 | 3200.00 | 100.45795° | 37.05102° | 8.10±0.09 | 5.66±1.40 | 42.21±4.72 | 50.44±3.82 | 7.62±1.09 | 4.13±0.11 | 2.82±0.09 |
| Q7 | 3235.00 | 100.77217° | 36.76942° | 7.78±0.19 | 11.16±1.02 | 119.22±22.41 | 134.00±20.94 | 10.85±2.93 | 4.14±0.10 | 1.83±0.82 |
| Q8 | 3208.00 | 100.12031° | 36.62688° | 7.40±0.60 | 7.30±0.39 | 60.84±2.34 | 60.87±2.15 | 8.34±0.13 | 4.87±0.05 | 3.25±0.17 |
| Q9 | 3208.00 | 99.79803° | 36.71103° | 7.09±0.26 | 5.64±0.25 | 40.70±9.32 | 59.87±1.30 | 7.18±1.36 | 4.95±0.26 | 2.08±0.67 |
| Q10 | 3198.00 | 99.60417° | 36.94395° | 8.14±0.65 | 3.71±0.59 | 20.27±9.44 | 66.53±11.33 | 5.48±2.23 | 5.81±0.28 | 1.38±0.47 |
| Q11 | 3460.00 | 99.36034° | 37.42461° | 8.33±0.09 | 4.74±0.33 | 44.06±4.88 | 145.07±23.92 | 9.27±0.37 | 4.53±0.03 | 1.67±0.39 |
| Q12 | 2861.00 | 97.24731° | 37.19988° | 8.18±0.29 | 3.92±0.46 | 29.43±7.52 | 70.96±4.92 | 7.44±1.40 | 7.21±2.56 | 0.88±0.41 |
| Q13 | 2850.00 | 97.57829° | 37.13914° | 7.96±0.78 | 14.35±2.66 | 155.17±12.92 | 46.43±4.24 | 10.98±1.40 | 4.73±0.03 | 5.21±0.44 |
| Q14 | 2822.00 | 96.85192° | 37.33516° | 8.43±0.31 | 4.13±0.28 | 21.67±2.66 | 76.13±2.60 | 5.29±0.96 | 4.43±0.28 | 1.35±0.07 |
| Q15 | 2844.00 | 99.4311° | 37.19139° | 8.11±0.18 | 0.58±0.05 | 3.25±0.72 | 56.72±9.98 | 5.55±0.83 | 3.99±0.23 | 0.24±0.01 |
| Q16 | 2800.00 | 94.37537° | 37.98627° | 8.02±0.14 | 2.92±0.79 | 7.85±1.01 | 51.68±6.95 | 2.87±1.07 | 5.14±1.12 | 1.33±0.44 |
| Q17 | 2764.00 | 94.12305° | 38.36611° | 7.70±0.39 | 3.29±1.32 | 31.22±19.65 | 148.52±21.11 | 8.80±2.75 | 4.65±0.35 | 0.32±0.14 |
| Q18 | 2804.00 | 94.12052° | 38.92691° | 7.37±0.30 | 7.59±0.80 | 100.54±18.54 | 73.64±14.51 | 13.17±1.15 | 4.15±0.12 | 2.19±0.51 |
| Q19 | 2745.00 | 93.34128° | 37.87989° | 8.05±0.58 | 4.45±1.27 | 66.54±12.66 | 201.30±33.47 | 15.63±4.47 | 5.07±0.41 | 1.13±0.34 |
| Q20 | 2864.00 | 90.65631° | 38.21865° | 9.25±0.38 | 1.73±0.84 | 24.06±2.61 | 21.68±4.63 | 15.36±4.64 | 5.40±1.47 | 1.23±0.24 |
| Q21 | 2878.00 | 91.72157° | 37.8147° | 7.48±0.17 | 3.85±0.71 | 70.31±5.64 | 66.03±18.28 | 18.55±2.64 | 9.70±11.11 | 2.22±0.76 |
| Q22 | 2717.00 | 96.24637° | 36.48832° | 6.99±0.63 | 6.03±0.91 | 144.30±14.44 | 131.12±9.56 | 24.11±2.35 | 5.04±0.95 | 0.52±0.13 |
| Q23 | 4402.00 | 94.21987° | 35.06667° | 7.63±0.34 | 12.63±3.24 | 243.73±18.56 | 172.88±25.95 | 20.20±5.37 | 4.46±0.09 | 1.34±0.10 |
| Q24 | 4522.00 | 93.9916° | 35.09671° | 8.47±0.30 | 4.72±1.04 | 91.02±11.74 | 121.29±40.46 | 20.11±5.67 | 4.16±0.07 | 0.80±0.23 |
| Q25 | 4702.00 | 92.05907° | 33.6435° | 8.31±0.12 | 2.86±0.29 | 62.31±2.92 | 85.17±31.09 | 21.89±1.26 | 4.33±0.08 | 1.04±0.31 |
| Q26 | 4444.00 | 94.73296° | 34.92972° | 7.81±0.31 | 4.12±0.11 | 85.93±4.31 | 86.89±11.79 | 20.87±0.54 | 4.54±0.12 | 1.78±0.30 |
| Q27 | 4530.00 | 94.87524° | 34.269° | 7.74±0.36 | 2.42±0.66 | 19.33±0.34 | 23.76±0.01 | 8.31±1.84 | 4.28±0.21 | 2.10±0.45 |
| Q28 | 4622.00 | 95.32583° | 33.80722° | 7.82±0.52 | 3.37±0.23 | 72.37±6.04 | 43.62±6.33 | 21.50±2.12 | 4.39±0.12 | 2.44±0.14 |
| Q29 | 4364.00 | 96.08402° | 33.51455° | 7.70±0.65 | 6.95±0.35 | 136.74±12.26 | 123.33±31.65 | 19.68±1.60 | 4.34±0.02 | 1.74±0.23 |
| Q30 | 4796.00 | 96.24332° | 33.35003° | 7.03±0.43 | 8.17±1.38 | 239.78±94.31 | 103.07±58.45 | 28.64±6.52 | 4.36±0.13 | 1.87±1.38 |
| Q31 | 4435.00 | 97.27861° | 33.59361° | 7.27±0.39 | 10.56±1.85 | 185.68±9.28 | 163.22±3.10 | 17.87±2.56 | 4.23±0.17 | 1.46±0.26 |
| Q32 | 4491.00 | 97.2523° | 33.88937° | 7.83±1.07 | 7.49±1.42 | 137.83±65.94 | 111.15±35.36 | 17.81±5.11 | 4.32±0.05 | 0.92±0.11 |
| Q33 | 4613.00 | 97.77898° | 34.17992° | 7.37±0.48 | 9.63±1.12 | 160.39±7.30 | 187.25±13.88 | 16.75±1.36 | 4.38±0.01 | 1.45±0.17 |
| Q34 | 4324.00 | 96.8766° | 35.06485° | 8.55±0.33 | 1.48±0.76 | 32.55±7.74 | 36.30±6.14 | 23.75±5.51 | 4.48±0.33 | 1.07±0.29 |
| Q35 | 4345.00 | 97.95361° | 34.84611° | 8.06±0.32 | 1.79±0.18 | 29.14±4.70 | 50.29±10.62 | 16.45±3.24 | 4.40±0.09 | 2.01±0.28 |
| Q36 | 4693.00 | 99.04361° | 34.81955° | 7.05±0.13 | 7.33±1.40 | 115.09±13.81 | 48.25±2.38 | 16.28±4.74 | 5.17±0.38 | 2.92±0.44 |
| Q37 | 4509.00 | 99.11138° | 34.69426° | 8.22±0.92 | 6.20±0.28 | 100.78±6.47 | 161.59±3.99 | 16.27±0.81 | 4.33±0.09 | 0.80±0.18 |
| Q38 | 4123.00 | 100.05933° | 33.48335° | 7.20±0.18 | 9.89±0.86 | 148.12±17.89 | 80.06±3.29 | 15.13±2.90 | 4.54±0.03 | 2.06±0.59 |
| Q39 | 3973.00 | 100.97297° | 33.40646° | 7.32±0.25 | 9.49±1.72 | 244.39±35.59 | 173.61±8.01 | 26.29±5.47 | 4.79±0.08 | 2.16±0.06 |
| Q40 | 3436.00 | 101.41194° | 35.02694° | 7.46±0.38 | 5.90±0.42 | 117.27±3.43 | 46.14±1.26 | 19.93±1.35 | 4.75±0.04 | 2.16±0.18 |
| Q41 | 3433.00 | 96.19305° | 32.52944° | 6.29±0.25 | 16.73±2.30 | 345.69±24.11 | 199.53±12.27 | 20.99±3.70 | 5.57±0.26 | 1.68±0.10 |
| Q42 | 4357.00 | 96.29554° | 32.14667° | 6.42±0.11 | 17.35±2.64 | 317.66±13.36 | 239.82±10.28 | 18.6±2.99 | 6.30±0.01 | 1.37±0.07 |
| Q43 | 4875.00 | 92.14916° | 33.06611° | 7.76±0.17 | 16.22±1.22 | 282.08±8.97 | 132.87±28.99 | 17.48±1.73 | 5.04±0.08 | 3.85±0.08 |

Supplementary Table S2 – Number of bacterial and fungal orders within phyla with positive or negative responses to each environmental variable.

| Phyla | Elevation | | pH | | TC | | TN | | C:N | | DOC | | E2/E3 | | SUVA254 | |
| --- | --- | --- | --- | --- | --- | --- | --- | --- | --- | --- | --- | --- | --- | --- | --- | --- |
|  | Freq+ | Freq- | Freq+ | Freq- | Freq+ | Freq- | Freq+ | Freq- | Freq+ | Freq- | Freq+ | Freq- | Freq+ | Freq- | Freq+ | Freq- |
| *Acidobacteria* | 2 |  |  |  |  | 2 | 2 | 1 |  | 2 |  | 2 |  | 3 |  | 1 |
| *Actinobacteria* | 3 |  |  | 8 | 3 |  | 1 | 2 |  | 2 |  |  |  | 5 | 4 |  |
| *Aminicenantes* |  |  |  |  | 1 |  |  |  |  |  |  |  |  |  |  | 1 |
| *Bacteroidetes* | 1 |  |  | 3 | 3 |  |  |  |  | 3 |  |  |  | 2 | 1 | 1 |
| *Chlorobi* | 1 |  |  |  |  |  |  |  |  | 2 |  | 1 |  | 1 |  | 1 |
| *Chloroflexi* | 2 |  |  | 2 |  |  |  |  |  | 2 |  |  |  | 4 | 1 |  |
| *Deinococcus–Thermus* |  | 1 |  |  |  |  |  |  |  |  |  |  |  |  |  |  |
| *Fibrobacteres* |  |  |  |  |  |  |  |  |  | 1 |  |  |  |  |  |  |
| *Firmicutes* | 3 | 1 |  | 1 |  |  |  | 1 |  | 1 | 1 |  | 1 |  | 1 |  |
| *Gemmatimonadetes* |  |  |  |  |  |  |  |  |  | 8 |  | 1 |  |  |  |  |
| *Latescibacteria* |  |  |  |  |  |  |  |  |  | 4 |  | 1 |  |  |  |  |
| *Nitrospirae* |  |  | 1 |  |  |  |  |  |  | 2 |  |  |  |  |  |  |
| *Proteobacteria* | 2 | 2 | 4 | 9 | 1 | 1 | 2 |  | 2 | 1 |  | 5 | 1 | 11 |  | 1 |
| *Thermomicrobia* |  |  |  | 1 |  |  |  |  |  |  |  |  |  |  |  |  |
| *Ascomycota* | 5 | 1 | 4 | 6 | 2 | 5 | 6 | 2 | 1 |  | 6 | 5 | 1 | 1 | 5 | 2 |
| *Basidiomycota* | 1 | 1 | 1 | 5 | 2 | 1 | 4 | 1 |  |  |  |  |  |  |  |  |
| *Glomeromycota* |  |  | 1 |  |  |  | 1 |  |  |  |  |  | 1 |  |  |  |
| *Zygomycota* | 1 | 1 | 1 |  |  |  |  |  |  |  |  | 1 |  |  |  |  |

Supplementary Figure S1. Ranked point estimates with 95% confidence intervals for pH (left panel) and elevation (right panel) based on the negative binomial LVM. Only microbial taxa for which the HPD interval did not cross zero are shown.

Supplementary Figure S2. Ranked point estimates with 95% confidence intervals for total C (left panel) and total N (right panel) based on the negative binomial LVM. Only the microbial taxa for which the HPD interval did not cross zero are shown.

Supplementary Figure S3. Ranked point estimates with 95% confidence intervals for the C:N ratio (upper left), dissolved organic carbon (upper right), E2/E3 ratio (bottom left panel), and SUVA254 (bottom right panel) based on the negative binomial LVM. Only the microbial taxa for which the HPD interval did not cross zero are shown.

Supplementary Figure S4: P

Supplementary Figure S5 – Positive shifts in bacteria-bacteria interactions following the changes in the elevation of the Qinghai-Tibet plateau.

Supplementary Figure S6 – Negative shifts in bacteria-bacteria interactions following the changes in the elevation of the Qinghai-Tibet plateau.

Supplementary Figure S7 – Positive shifts in bacteria-bacteria interactions following the changes in the soil C:N ratio of the Qinghai-Tibet plateau.

Supplementary Figure S8 – Negative shifts in bacteria-fungi interactions following the changes in the soil C:N ratio of the Qinghai-Tibet plateau.
